# “I Can Sense When My Hands Need Washing”: A Qualitative Study and Thematic Analysis of Factors Affecting Young Adults’ Hand Hygiene

**DOI:** 10.1177/11786302221129955

**Published:** 2022-10-15

**Authors:** Abhinand Thaivalappil, Ian Young, David L Pearl, Jennifer E McWhirter, Andrew Papadopoulos

**Affiliations:** 1 Department of Population Medicine, University of Guelph, ON, Canada; 2 School of Occupational and Public Health, Toronto Metropolitan University, ON, Canada

**Keywords:** COVID-19, hand hygiene, health behavior, thematic analysis, qualitative methods, young adults

## Abstract

Handwashing is one of the most effective and low-cost public health measures. However, it is often not practiced frequently enough or correctly by the public. Young adults in particular have poorer intentions to wash their hands, frequency of handwashing, and sanitizer use compared to other age groups. Therefore, there is a need to identify barriers and facilitators affecting hand hygiene within this group. The objective of this qualitative study was to apply the Theoretical Domains Framework to explore factors which influence hand hygiene among young adults aged 18 to 25 years old. An online questionnaire (n = 37) and thematic analysis were used to generate 3 overarching themes. The main findings indicated internal factors such as knowledge and intentions; interpersonal factors such as social norms; and environmental factors such as reminders, cues, accessibility, and cleanliness of handwashing facilities determined the level of hand hygiene practiced among young adults. The findings suggest that behavior change techniques such as social comparisons and tailored messaging to suit the needs of young adults may be more effective at increasing hand hygiene.

## Introduction

The coronavirus disease 2019 (COVID-19) pandemic resulted in governments mobilizing in numerous ways to curb virus transmission by promoting and implementing public health interventions, many of which have been effective.^1-3^ One such intervention was promoting hand hygiene, where handwashing recommendations were reiterated and efforts were made to increase access to handwashing facilities and alcohol-based hand sanitizer.^4-7^ Despite this, adherence to handwashing recommendations have varied during the COVID-19 pandemic.^8^ In the early stages of the pandemic, members of the public and healthcare providers had increased hand hygiene frequency and compliance, but this was short-lived and there was a decline to baseline levels over time.^9,10^ Although evidence now suggests that infection through contact by contaminated surfaces is not a primary source of COVID-19 infection, hand hygiene remains important in preventing community transmission of various respiratory and enteric infections.^11-13^

Hand hygiene adherence is an ongoing issue among the public and healthcare workers.^9,14^ Despite ample evidence demonstrating handwashing reduces the risk of gastrointestinal and respiratory illnesses, reduces hospital-acquired infections, reduces absenteeism, and improves child development,^15^ compliance across different groups has been relatively low.^14,16^ One such group of particular interest is young adults. Although the definition varies across the literature, some research studies have defined young adults as those aged 18 to 25 years due to their unique psychosocial and health needs, and the major transitions they undergo from adolescence to adulthood.^17-19^ Regarding hand hygiene, previously published research have found self-reported hand hygiene to be lower among young adults compared to other age demographics.^20-22^Moreover, young adults have reported lower intentions to practice handwashing compared to other age groups ^23^; young adults aged 18 to 29 had a lower frequency of washing and sanitizing hands compared to other age groups ^10,24^; and there is evidence to support these behaviors were similar prior to the pandemic suggesting this demographic is unique and young adults face additional barriers to hand hygiene.^25,26^ Studies investigating college and college-aged students have found high variability in self-reporting of handwashing, ranging between 7% and 88%.^27-30^ However, criticisms of self-report measures in assessing handwashing exist and observational studies have found hand hygiene to be much different, ranging between 26% and 62%.^26,31-33^ Both types of studies indicate young adults may benefit from interventions promoting hand hygiene and these interventions may be more effective when tailored to a specific age demographic.

The United Nations Children’s Fund^4^ has stated the dissemination of knowledge is insufficient in prolonged behavior change and called for tailored interventions which incorporate items which groups are invested in and include relevant social norms, motivators, and emotions. Furthermore, in a systematic review of hand hygiene improvement, Huis et al^34^ indicated multiple approaches which address barriers to change, along with internal, interpersonal, and organizational factors may be needed to change handwashing behaviors. Quantitative researchers exploring handwashing behaviors and barriers have provided insights for creating targeted behavior change interventions and some of these researchers have applied behavior change theories to guide their research.^23,35-38^ Additionally, some qualitative researchers have also investigated hand hygiene practices in other groups besides young adults.^39,40^ However, there is a need to explore drivers of hand hygiene practices among young adults aged 18 to 25 years and provide a snapshot of current barriers to handwashing. This investigation may provide clues to developing tailored health messages and behavior change interventions among this group of adults. Therefore, the aim of this qualitative study was to identify the barriers and facilitators to hand hygiene among young adults aged 18 to 25 years old.

## Methods

### Study design

This study utilized a qualitative descriptive approach,^41,42^ which emphasizes staying close to the data to provide a comprehensive summary of ideas and events.^43^ Qualitative description was selected because of the flexibility the design offers in its use of methods and techniques, and it presented the best fit between theoretical underpinnings of the research as well as the analytic approaches used in this study. Reporting guidelines followed the Standards for Reporting Qualitative Research.^44^

The study was designed with feedback from young adults aged 18 to 25 and public health experts. We applied the Theoretical Domains Framework (TDF) as a lens to guide the design, data collection, analysis, and reporting. The TDF contains 14 theoretical domains synthesized originally from 33 behavior change theories which covers 128 constructs.^45,46^ The framework is intended to assist in exploring and categorizing drivers of health behavior and to enhance implementation science.^47^ It has been previously applied in quantitative and qualitative research investigating hand hygiene and other behaviors,^48-52^ and these were used to inform the current study.

An online qualitative survey design was used rather than traditional interviews or focus groups because of the unobtrusive nature, flexibility, and non-direct contact with respondents. Online surveys, if designed appropriately, offer numerous benefits in conducting qualitative research.^53^

### Participants and recruitment

This study was conducted entirely online and was posted internally within STEM Fellowship, which is a Canadian-based, volunteer-led, charity organization aimed at providing science, technology, engineering, and mathematics opportunities to students across the country. At the time of data collection in January 2022, there were 320 members within STEM Fellowship. The organization invites volunteers from across the country who are mostly high school and university students to host and participate in STEM workshops, webinars, competitions, and bootcamps.

A recruitment ad was created and shared across the organization’s channels along with a link to the online questionnaire. The questionnaire was administered using Qualtrics (Provo, Utah, US), an online survey software platform. Written consent was obtained at the beginning of the questionnaire. The questionnaire was only available in English and to those who were aged 18 to 25 years old. Upon completion of the questionnaire, respondents were offered a $10 e-gift card of their choice of Amazon, Apple, or Best Buy.

### Positionality

AT was a member of STEM Fellowship and therefore had existing relationships with other members. Numerous steps were taken to ensure participation in the study had no impact on the working relationship between members within STEM Fellowship. All authors come from a public health or epidemiology background and have been involved in disease prevention and health promotion initiatives and research. AT and IY have previous health behavior expertise in hand hygiene, and all others were public health experts who had investigated the young adult demographic regarding other topics.

### Materials and procedure

Questions were adapted from a previous study outlining the implementation of the Theoretical Domains Framework (TDF) for qualitative research.^48^ This was modified to fit the topic of interest in this study and included additional factors of interest (eg, culture, government). In total, the questionnaire consisted of 15 items: 2 on demographics (age and gender), and 13 open-text questions asking about various factors related to handwashing (eg, How easy, or difficult do you find acting on these recommendations?). The full questionnaire is available as supplementary material.

The questionnaire explored young adults’ attitudes, beliefs, and general perspectives toward handwashing with an emphasis on the COVID-19 pandemic. An infographic from the Government of Canada was also shared within the questionnaire and is provided as supplementary material. All questions were reviewed by members of the research team, and pilot tested by 3 members of STEM Fellowship who were within the target age range of 18 to 25 years old. The pilot test phase included asking the individuals to complete the survey once, and provide the first author with feedback on readability, survey length, and redundancy of questions. This process resulted in the rephrasing, deletion, and merging of questions until all parties agreed through discussion and approved the final questionnaire. All data were collected in January 2022.

### Ethical approval

No identifying information about the participants were collected and no direct contact were made between the researchers and participants unless the participants indicated they wished to be contacted for member checking, described below. All participants were asked to read a consent form, agree to participate, and declare they were between the aged of 18 and 25 before commencing the questionnaire. Any individual outside of the age range and those who did not consent were redirected to the end of the survey. Ethical approval was granted by the Research Ethics Board at the University of Guelph (REB#21-10-026).

### Data analysis

The analysis was conducted from a contextual constructionism epistemological position.^54^ We employed a semantic thematic analysis using a predominantly deductive or theory-driven approach as this research was guided by the TDF. A semantic approach involves identifying themes from the surface meaning of the data rather than aiming to go beyond the semantic content to identify the underlying assumptions or ideas.^55^ Although there was an emphasis on semantic approaches, this research was underpinned by our theoretical assumptions and used interpretive approaches to construct meanings by combining respondents’ experiences with our own interpretations. Therefore, we heavily leaned on reflexive thematic analysis which moves away from positivist values (eg, inter-reliability measures, codebook development) and instead embraces qualitative research values stating coding can be open, be inductive or deductive, and is situated within interpretive reflexive methods to generate themes using an iterative process.^56^

After multiple readings of the qualitative responses and undergoing the data familiarization process, additional codes were inductively generated through discussion between the research team members to encompass factors which were not fully captured by the TDF. Following coding, the most relevant theoretical domains were selected based on 2 criteria: (a) the frequency of specific beliefs reported, and (b) any indication of strong beliefs which affected handwashing practices among respondents. Stronger beliefs were demonstrated when individuals reported fewer differences between respondents, and when written responses expressed conviction and were free of doubts. Underlying belief statements were collated, and overarching themes were generated from these salient domains. Accounts were revisited multiple times by AT to ensure themes were supported by the data. These were discussed between AT and IY to revise the sub-themes and themes.

Two forms of triangulation were used in this study. First, researcher triangulation in the form of peer debriefing and discussions were conducted throughout the analysis to generate richer interpretations of data. This was complemented with member checking, where respondents were followed-up with and invited to provide written feedback on the overall findings. Both approaches were used to aim for reflexivity and collaboration rather than with the purpose to reach a consensus.^57^

Data analysis was conducted using NVivo 1.6.1 qualitative analysis software (QSR International, Doncaster, Australia). All steps were conducted by AT and reviewed by IY through discussion.

## Results

Overall, 37 individuals participated in the questionnaire and the demographics are listed in Table 1. Only one submitted an incomplete survey. Most respondents were between 18 and 23 years old, one respondent was 24 years old, none were 25 years old, and one individual preferred not to declare their age (Table 1). Several salient domains were identified: (1) environmental context and resources; (2) behavioral regulation; (3) knowledge; (4) memory, attention, and decision processes; (5) social influences; (6) intentions; and (7) social role and identity. Two additional categories were also identified to be relevant and recurring: (1) government, and (2) society and culture. Sub-themes and themes were generated from the data and relied heavily on the predominant domains. These findings are described below, and additional exemplar quotes can be accessed as supplementary material.

**Table 1.** Respondents’ demographic characteristics in the hand hygiene questionnaire conducted in January 2022 (n = 37).

Characteristic	n (%)
Age:	
18	7 (18.9)
19	11 (29.7)
20	7 (18.9)
21	5 (13.5)
22	2 (5.4)
23	3 (8.1)
24	1 (2.7)
Prefer not to say	1 (2.7)
Gender:	
Man	17 (45.9)
Woman	19 (51.4)
Non-binary/third gender	1 (2.7)

### Theme 1: An inherent responsibility with some flexibility on the recommendations followed

Four domains were grouped under this theme: knowledge, individual role and identity, behavioral regulation, and intentions. Most young adults were aware of the recommendations and felt comfortable and confident following them. Some admitted they followed recommendations closely with some deviation in certain steps described.

*“[T]hat is exactly how I wash my hands. However, I tend to close the tap with my elbow instead of a paper towel and my hand.”* (P9, female, 19 years old)

Respondents felt it was their responsibility to have good hand hygiene as well as others’ responsibility to do the same:

*“Each person should carry the responsibility of proper handwashing in order to reduce the spread of germs.”* (P1, female, 20 years old)

Respondents were confident in their ability to follow recommendations because handwashing had become a habit instilled in them since a young age.

*“I can sense when my hands need washing, which I do quite often and it occurs naturally to me.”* (P4, male, 21 years old)

Most young adults believed good handwashing was easy to perform and they indicated doing so. However, there were also several respondents who also expressed they did not follow the recommendations.

*“I have the ability to but I don’t think I would want to.”* (P24, non-binary/third gender, 24 years old)

### Theme 2: Time and location as key contributors to handwashing

Two domains were grouped under this theme: environmental context and resources; and memory, attention, and decision processes. Handwashing recommendations were not followed fully because of time, location, and lack of resources.

*“I would likely be even more stringent at all times in times where infections (e.g., COVID, flu season) are notably trending upwards in my vicinity or if I felt like the environments and activities around me are less clean/more crowded.”* (P6, female, 23 years old)

*“Sometimes there isn’t paper towel to turn the tap off so you need to do it with your hands instead, and then use a dryer to dry them.”* (P15, male, 18 years old)

Many individuals cited internal and external cues to action which enabled them to wash their hands or served as a reminder. Some indicated that more reminders may be necessary, but there was a small minority who questioned the effectiveness of signs:

*“I wash my hands after I go outside, before eating, in the washroom, and any time I touch something that is ‘dirty’.”* (P3, female, 20 years old)

*“I would need a sign like this posted at every sink that I wash my hands at, only because I usually forget to do this.”* (P37, male, 18 years old)

### Theme 3: A social norm which is encouraged by all

Three categories were grouped under this theme: government credibility and health messaging; social and organizational influences; and societal and cultural pressures. Many respondents appreciated government’s actions regarding handwashing interventions, but some concerns were also raised:

*“Society definitely makes me feel obligated to practice good hand hygiene and instructions from government officials and public health professionals help remind me of the importance of good hand hygiene.”* (P7, female, 22 years old)

*“. . .just posting signs isn’t enough from the government - they need to ensure that everyone understands the importance of washing your hands, as well as has access to clean water.”* (P14, male, 18 years old)

Respondents commented that people around them practiced good hand hygiene. Most also acknowledged peers and employment influenced their handwashing practices:

*“I think that my long-term care [home] work culture certainly helps with practicing good hand hygiene. Every year, we watch training videos on infection prevention and control measures, including handwashing.”* (P36, female, 21 years old)

Young adults generally reported their community and culture had a large impact on facilitating good hand hygiene:

*“These guidelines have been taught in my schools growing up.”* (P18, female, 20 years old)

## Discussion

We explored the barriers and facilitators toward hand hygiene practices among young adults using qualitative approaches which aim for value-laden, context-dependent thick and rich description. Findings from the thematic analysis revealed several barriers and facilitators for good hand hygiene practices among young adults involved in STEM initiatives across Canada. The 3 overarching themes generated were: an inherent responsibility with some flexibility on the recommendations followed; time and location as key contributors to handwashing; and a social norm which is encouraged by all.

There was an equal balance between internal motivators, interpersonal factors, and environmental pressures on hand hygiene practices within this group. Among internal factors, there were some gaps in intentions, knowledge, and memory. There were steps within handwashing which were new to some respondents, reports of low intentions, and forgetfulness to practice handwashing. Knowledge has been associated with stronger hand hygiene practices,^58,59^ but this relationship may be weak as one study showed significant differences between knowledge and practice among young adults.^60^ An effective behavior change technique (BCT) to improve knowledge and intentions is to provide information about health consequences.^61^ Results from the present study revealed that the one respondent who had work experience in healthcare reported stronger intentions and better self-reported handwashing habits, which could be due to being more informed about health consequences of noncompliance. A previously published study found over 60% of respondents reported forgetting to wash their hands.^60^ This was found, to a lesser degree, in the current study and does suggest prompts and cues in the form of signage may be needed to increase and maintain handwashing frequency among young adults.^61^ Prompts and reminders can be effective, and previous research argues the importance of developing them with care because of variability in effectiveness of these interventions.^62-64^ Thus, it may be appropriate to tailor reminders to focus on gaps such as 1 or 2 aspects of handwashing (eg, scrubbing underneath the nails, rubbing thumbs) in settings with predominantly young adults such as universities and college campuses.

Normative influences were a clear motivator for respondents’ own handwashing practices. Most respondents reported highly of their peers’ and communities’ hand hygiene, and it suggests descriptive norms were already at a high level among this group. There were some challenges in determining which norms prevailed, but the findings from this study hint injunctive norms could be targeted to improve handwashing frequency and duration. In the theory of normative social behavior, injunctive norms are expectations of others’ beliefs regarding the acceptability or unacceptability of a behavior^65^ and it is analogous to the Theory of Planned Behavior (TPB) subjective norms. These norms are theorized to moderate the effect of descriptive norms on behaviors,^65^ and normative factors have shown to influence handwashing behaviors.^66^ Interventions focusing on social comparisons and information about other’s approval could be effective in changing behaviors and establishing behavior change maintenance.^61,67^ Similarly, there were responses from this study indicating self-image played a role in hand hygiene. This was highlighted following data collection where one respondent communicated to the first author on the possibility of exaggerated responses, suggesting there were socially desirable responses. Social desirability and overreporting of handwashing have been found,^59,68^ and another qualitative study among student nurses also found similar sentiments between handwashing and the importance of self-image.^69^ Therefore, self-image and message framing may be an avenue for future qualitative investigations because we were unable to form any conclusions due to the limited data collected concerning this topic in our study.

The most predominant ecological determinants of hand hygiene practices were related to the physical environment and adequate time. Respondents not only mentioned signage, but also access to clean facilities, facilities which were not crowded, resources such as soap and hand sanitizer, and not feeling rushed as key factors which influenced whether they practiced good handwashing. Time was often mentioned as a barrier to practicing good hand hygiene, and respondents reported skipping steps to save time to prioritize other tasks. Similar barriers have been outlined by healthcare workers in a hospital setting.^70^ In the case of time, it may be beneficial to encourage the use of alcohol-based hand sanitizers as a substitute in low-compliance areas. The physical environment was the most frequently mentioned barrier to hand hygiene found in this study. Not all handwashing facilities are the same. Some have air dryers, motion-sensing faucets, and swinging-type exit doors which are pushed open (vs traditional doorknobs or handles). It is optimal to restructure the environment to promote hygienic behaviors and reduce opportunity costs,^61,71^ but this is not always feasible. Cost-effective solutions such as tailoring signage and messaging to specific areas may ensure individuals do not rely on self-regulation or are impacted by lapses that lead to behavior change maintenance failure.^71^ Moreover, the literature recommends multimodal approaches to increase handwashing frequency and duration.^4,14,59^ Specifically, infrastructure-related interventions in combination with signage and messaging, such as reminders, may be most effective.^14,72^ We echo these recommendations and call for multimodal approaches to handwashing interventions.

## Strengths and Limitations

This study used a validated theoretical framework to assess the barriers and facilitators to good hand hygiene practices among young adults. Its aim was to improve our understanding of behavior change processes and enhance implementation of evidence-based practice.^45,46^ Additionally, approaches to enhance the trustworthiness of the findings were used through researcher triangulation and member checking.^73^ The first author had also previously developed relationships with many of the respondents, and reflexivity was easier to exercise. We acknowledge this may have introduced additional bias into the engagement of respondents and resulting dialog, but many attempts were made to have the first author removed from this process such as using an online and anonymous questionnaire. Respondents were given a choice to be contacted upon completion of the questionnaire as well. We believe the researcher-participant relationship contributed to thick and rich descriptions of the data.

This study was not without limitations. We were unable to recruit a meaningful number of participants between 24 and 25 years of age, suggesting that increased efforts may be needed (eg, greater incentives) to recruit these individuals. We attempted to strike a balance between qualitative research values and implementation science by compiling recommendations for messaging and interventions aided by previous research. These findings are not meant to be generalizable or extended to wider populations because of the niche demographic being studied. Furthermore, we acknowledge this work was situated within qualitative research paradigms. Thus, more research is needed to investigate this topic to determine whether results are similar or different across different groups and contexts. Next, the median response time of the questionnaire was 10 minutes despite pilot testing showing a median response time of 25 minutes. This may have been exacerbated by using an optional response-type questionnaire and a non-controversial research topic. This suggests hand hygiene may not be a priority for young adults based on the lack of interest and rushed format of the questionnaire. Qualitative studies using online questionnaires may benefit from using fewer questions and reflection-type questions (vs standard structured questions) to reduce survey fatigue.^53^ Furthermore, our study only collected age and gender under demographic information, and researchers interested in collecting more demographic characteristics using similar survey approaches may have to opt for fewer qualitative-based questions to account for survey length and fatigue. Finally, we acknowledge the selection of relevant or predominant domains may have affected the generation of themes similar to a previous study using the same methodological approaches.^48^ However, we attempted to reduce this by including as many domains as possible during thematic analysis and only omitted ones that had limited data and those that were not considered to be salient factors for hand hygiene.

## Implications for Future Research

Several areas for future research were identified. First, this study was conducted in January 2022 when the COVID-19 pandemic and its associated risk-mitigation strategies aimed at the public were highly active. Thus, a repeat study in the future is recommended to determine whether the course of the pandemic and levels of health messaging have affected barriers and facilitators toward hand hygiene among young adults. Specifically, religious norms on hand hygiene practices, exploring barriers among lower-socioeconomic status young adults, and an evaluation of existing health messaging on handwashing. Qualitative studies exploring the impacts of religious norms on behavior could be important to separate and distinguish between cultural and religious barriers or facilitators. Identifying the level of influence religious norms have on health behaviors may help develop more appropriate messaging for young adults. Further, the study population for this investigation was recruited from STEM Fellowship and these individuals are generally of higher socioeconomic status and education level than other young adults in Canada. Thus, the respondents may have been more knowledgeable about hand hygiene than others. However, it is difficult to ascertain because in-depth demographic information beyond age and gender were not collected. Therefore, it may be beneficial to replicate this study in a lower socioeconomic young adult group to corroborate findings, and collect more demographic information (eg, education, household income). Caution is advised to keep surveys limited in length. Lastly, and although unintentional, respondents often commented on the handwashing infographic which was provided in the questionnaire, and individuals identified its strengths, weakness, and needs. This was not the purpose of the current study. However, these comments hinted there were areas for improvement in handwashing messaging and illustrations. Thus, it may be beneficial to compare several types of handwashing messaging with this demographic to assess areas for improved uptake.

## Conclusion

This qualitative study used the TDF as a lens along with thematic analysis to identify barriers and facilitators toward good hand hygiene practices among young adults. We found respondents had an inherent responsibility to follow good hand hygiene, were influenced by time, resources, and location, and perceived handwashing as a social norm which was encouraged by friends, family, and employment. Interventions aimed at improving hand hygiene in this population may benefit by implementing cues and reminders throughout relevant spaces, distilling messages to only include 1 or 2 low-compliant steps in handwashing, and tailoring messages to integrate BCTs such as social comparisons. Further research is strongly recommended to explore religious norms on hand hygiene and a similar investigation among young adults from lower socioeconomic backgrounds.

## Supplemental Material

sj-docx-1-ehi-10.1177_11786302221129955 – Supplemental material for “I Can Sense When My Hands Need Washing”: A Qualitative Study and Thematic Analysis of Factors Affecting Young Adults’ Hand Hygiene Click here for additional data file. Supplemental material, sj-docx-1-ehi-10.1177_11786302221129955 for “I Can Sense When My Hands Need Washing”: A Qualitative Study and Thematic Analysis of Factors Affecting Young Adults’ Hand Hygiene by Abhinand Thaivalappil, Ian Young, David L Pearl, Jennifer E McWhirter and Andrew Papadopoulos in Environmental Health Insights

sj-docx-2-ehi-10.1177_11786302221129955 – Supplemental material for “I Can Sense When My Hands Need Washing”: A Qualitative Study and Thematic Analysis of Factors Affecting Young Adults’ Hand Hygiene Click here for additional data file. Supplemental material, sj-docx-2-ehi-10.1177_11786302221129955 for “I Can Sense When My Hands Need Washing”: A Qualitative Study and Thematic Analysis of Factors Affecting Young Adults’ Hand Hygiene by Abhinand Thaivalappil, Ian Young, David L Pearl, Jennifer E McWhirter and Andrew Papadopoulos in Environmental Health Insights
