# Supplementary material for: “I Can Sense When My Hands Need Washing”: A Qualitative Study and Thematic Analysis of Factors Affecting Young Adults’ Hand Hygiene
Source: Environ Health Insights. 2022 Oct 15;16:11786302221129955. doi: 10.1177/11786302221129955 (PMC9575434; doi:10.1177/11786302221129955)
Supplement: sj-docx-1-ehi-10.1177_11786302221129955 – Supplemental material for “I Can Sense When My Hands Need Washing”: A Qualitative Study and Thematic Analysis of Factors Affecting Young Adults’ Hand Hygiene [file sj-docx-1-ehi-10.1177_11786302221129955.docx]

**Supplementary File 1.** Questionnaire on the factors influencing safe hand hygiene practices during the time of the COVID-19 pandemic among students.

1. What is your gender identity? *(open-text response)*
2. What is your age? *(insert number)*

Take a moment to review the infographic below. It was posted by the Government of Canada and outlines good handwashing practices [here](https://www.canada.ca/content/dam/phac-aspc/documents/services/publications/diseases-conditions/coronavirus/covid-19-handwashing/covid-19-handwashing-en.pdf).

A series of questions will be posed over the next 3 sections. All responses have an open-text feature with no word limit, and you are encouraged to respond if you feel comfortable. Please explain your responses with as much detail as you can to help us understand the way people think.

1. ***Knowledge.*** How familiar were you with this? Was there something you did not know?
2. ***Social role and identity.*** Do you feel it is your job or duty to do this? Please explain. Do you believe it should be others job to follow these recommendations?
3. ***Skills.*** How easy or difficult do you find acting on these recommendations?
4. ***Beliefs about capabilities.*** How confident are you that you can follow these recommendations? What makes you feel this way?
5. ***Beliefs about consequences.*** What do you think will happen if you do this? What about if you decided not to wash your hands?
6. ***Motivation and goals.*** How much do you want to act on the recommendations? Are there any incentives, or things that would need to change for you to want to follow them more often?
7. ***Memory, attention, and decision processes.*** Is following these handwashing recommendations something you usually do?
8. ***Social influences.*** Do your friends, family, coworkers, or community practice good hand hygiene? Do you feel pressure from anyone to practice good hand hygiene?
9. ***Government.*** Do you think the government, through its announcements or rules, plays a role in whether or not you practice good handwashing?
10. ***Culture, society, and community.*** Do you feel your culture or the Canadian society in general help or hinder whether your practice good hand hygiene?
11. ***Environmental context.*** Do you feel your physical environment helps or hinders whether you practice handwashing?
12. ***Emotion.*** How do you feel about following these recommendations? Do your feelings at the time affect whether or not you wash your hands correctly?
13. ***Behavioural regulation.*** Are there things you need to do before you can wash your hands? Are there things that help to prompt you to do it?
14. Anything else which we haven’t covered? Please include your final thoughts and comments here.

This concludes the hand hygiene survey. Please enter your name and email below if you wish to receive a gift card. You will be given the choice of the following cards of $10 value: Amazon, Apple, or Best Buy.

Thank you for participating in the survey.

**References**

Lawton, R., Heyhoe, J., Louch, G., Ingleson, E., Glidewell, L., Willis, T. A., ... & Foy, R. (2015). Using the Theoretical Domains Framework (TDF) to understand adherence to multiple evidence-based indicators in primary care: a qualitative study. *Implementation science*, *11*(1), 1-16.

Government of Canada. Reduce the spread of COVID-19: Wash your hands infographic. Retrieved October 4, 2021, from <https://www.canada.ca/content/dam/phac-aspc/documents/services/publications/diseases-conditions/coronavirus/covid-19-handwashing/covid-19-handwashing-en.pdf>
