# Supplementary material for: “I Can Sense When My Hands Need Washing”: A Qualitative Study and Thematic Analysis of Factors Affecting Young Adults’ Hand Hygiene
Source: Environ Health Insights. 2022 Oct 15;16:11786302221129955. doi: 10.1177/11786302221129955 (PMC9575434; doi:10.1177/11786302221129955)
Supplement: sj-docx-2-ehi-10.1177_11786302221129955 – Supplemental material for “I Can Sense When My Hands Need Washing”: A Qualitative Study and Thematic Analysis of Factors Affecting Young Adults’ Hand Hygiene [file sj-docx-2-ehi-10.1177_11786302221129955.docx]

**Supplementary File 2.** Themes, domains or constructs, and accompanying illustrative quotes from the young adults’ hand hygiene qualitative study.

| **Theme** | **Domain or construct** | **Illustrative quote(s) on factors influencing young adults’ hand hygiene behaviours** |
| --- | --- | --- |
| An inherent responsibility with some flexibility on the recommendations followed | Knowledge | *“Very familiar. This is how I was first taught to wash hands. Similar but different.”* (P22, female, 19 years old)  *“I was pretty familiar with hand washing as I have been doing it since a young age. However I didn’t know of cleaning under the nails when hand washing.”* (P17, female, 19 years old) |
|  | Social role and identity | *“It is my responsibility to wash my own hands, and it is others responsibility to wash their hands. If everyone adhered to these guidelines by ensuring their own responsibility is met, that is the ethnically (sic) optimal scenario.”* (P4, male, 21 years old)  *“I believe that it is my own responsibility to wash my hands, since I hope to minimize the spread of bacteria or viruses.”* (P18, female, 20 years old) |
|  | Behavioural regulation | *“I am very confident that I can follow these recommendations as they are already part of my daily lifestyle.”* (P1, female, 20 years old)  *“Washing my hands is kind of second nature now.”* (P10, female, 18 years old) |
|  | Intentions | *“I am actually inclined to… The steps are all simple and straightforward to follow.”* (P5, male, 19 years old)  *“I'd rather do something else with the time.”* (P26, male, age not specified)  *“I find the instructions easy however I don’t take the time to wash my hands that throughly (sic).”* (P17, female, 19 years old)  *“I personally don’t feel pressure to do so, it is just something I want to do.”* (P20, male, 19 years old) |
| Time and location as key contributors to handwashing | Environmental context and resources | *“I am more diligent with hand washing when I am not in a rush and if I am about to eat.”* (P7, female, 22 years old)  *“Sometimes it might not be 20 seconds if I'm in a rush.”* (P15, male, 18 years old)  *“…with the pandemic, I have been more conscious of the surfaces I come in direct contact with and actively try to limit my direct contact with public surfaces, in addition to carrying wipes and hand sanitizer.”* (P1, female, 20 years old)  *“I think the greatest barrier to good hand washing practices would be a lack of resources in public washrooms and I would need to resort to hand sanitizer which I usually carry on my person.”* (P7, female, 22 years old)  *“The main obstacle I feel I face is that 1) I do not know off the top of my head what 20 seconds is. I either over or underestimate the time in my head 2) the bathroom facility I am using doesn't provide paper towel - dryer machine instead. When I exit the bathroom I am forced to touch the door handle with my hands or t shirt.”* (P2, male, 21 years old)  *“I generally already follow these steps when I wash my hands and don't plan on changing that routine. As well, if there isn't time/access to a sink and soap, the increased access to hand sanitizer stations in public helps me still follow these recommendations.”* (P6, female, 23 years old) |
|  | Memory, attention, and decision processes | *“In places where clean hands are a must (e.g., labs, hospitals) I tend to pay more attention to the cleaning of my hands.”* (P5, male, 19 years old)  *“Yes, the sight of a sink.”* (P10, female, 18 years old)  *“I've noticed that signage is not very effective at reminding me though.”* (P7, female, 22 years old)  *“Quite easy; except I tend to forget to rub my thumbs as indicated in the infographic.”* (P31, female, 20 years old) |
| A social norm which is encouraged by all | Government | *“I think that COVID guidelines are stricter in Canada vs the US, so I feel more pressure to be a good citizen and use better hand hygiene when I’m in Canada.”* (P16, female, 20 years old)  *“I’m very confident in the government's recommendations. I have a lot of faith in Health Canada, as it is one of the most prestigious bodies of government in it's (sic) field in the world.”* (P13, male, 22 years old)  *"Sometimes very strict/constant government messaging can really feel like a chore or fruitless.”* (P6, female, 23 years old)  *“I believe most people wash their hands when appropriate, however probably not as thoroughly as recommended by the Government of Canada. I don’t really feel pressure from anyone in particular to practice good hand hygiene.”* (P36, female, 21 years old) |
|  | Social and organizational influences | *“Practicing hand hygiene is a positive thing and I want to be seen in a positive light by those around me.”* (P10, female, 18 years old)  *“I think that many people think that having good hygiene is attractive, so that also pushes me to practice good hand hygiene.”* (P3, female, 20 years old)  *“Yes, the people around me practice good hand hygiene. I don’t feel pressure from anyone other than myself to practice good hand hygiene.”* (P25, female, 21 years old)  *“Most of my friends, family and coworkers practice good hand hygiene, however, I am not aware of my local community's hand hygiene practices. I do feel pressure/obligated from general societal pressures to practice good hygiene.”* (P7, female, 22 years old) |
|  | Societal and cultural pressures | *“I believe on social media in todays day in age, when someone sees a person not practicing proper hand hygiene, they may call them out on it, especially on popular apps such as Tiktok.”* (P33, female, 18 years old)  *“I believe my family's values and the general Chinese culture of following guidelines encourages me to practice good hand hygiene.”* (P34, female, 18 years old) |
